# Overexpression of *HPRT1* is associated with poor prognosis in head and neck squamous cell carcinoma

**DOI:** 10.1002/2211-5463.13250

**Published:** 2021-08-04

**Authors:** Mohsen Ahmadi, Maryam Eftekhari Kenzerki, Seyed Mohammad Akrami, Salar Pashangzadeh, Fatemeh Hajiesmaeili, Sahereh Rahnavard, Leila Habibipour, Negin Saffarzadeh, Pegah Mousavi

**Affiliations:** ^1^ Student Research Committee Hormozgan University of Medical Sciences Bandar Abbas Iran; ^2^ Department of Medical Genetics Faculty of Medicine Hormozgan University of Medical Sciences Bandar Abbas Iran; ^3^ Division of Medical Genetics Booali Medical Diagnostic Laboratory Qom Iran; ^4^ Department of Medical Genetics School of Medicine Tehran University of Medical Sciences Iran; ^5^ Iranian Research Center for HIV/AIDS Iranian Institute for Reduction of High‐Risk Behaviors Tehran University of Medical Sciences Iran; ^6^ Department of Cellular and Molecular Biology Ahar Branch Islamic Azad University Ahar Iran; ^7^ Molecular Medicine Research Center Hormozgan Health Institute Hormozgan University of Medical Sciences Bandar Abbas Iran; ^8^ Department of Nephrology Hasheminejad Kidney Center Iran University of Medical Sciences Tehran Iran

**Keywords:** bioinformatics, biomarker, GEO, head and neck cancer, HPRT1, TCGA

## Abstract

Hypoxanthine phosphoribosyltransferase (*HPRT1*), as a salvage pathway enzyme, plays a crucial role in modulating the cell cycle and has been reported to be overexpressed in multiple cancers. Nevertheless, the relationship between the *HPRT1* gene and head and neck squamous cell carcinomas (HNSCCs) has not been investigated so far. In this study, we first evaluated the expression and clinical value of *HPRT1* mRNA and protein in tumor and healthy control tissues. Then, we examined mutations of the *HPRT1* gene and their association with survival outcomes of patients with HNSCC. We also performed functional analyses of *HPRT1* coexpressed genes and examined the association between HPRT1 expression and drug sensitivity. Both *HPRT1* mRNA and protein were significantly higher in HNSCC compared with normal tissues, and up‐regulation of *HPRT1* was also correlated with age, sex, pathological stage and histological grades of patients with HNSCC. Moreover, *HPRT1* and its associated genes were observed to be enriched for several cancer‐related pathways, including DNA replication and cell cycle. Finally, patients exhibiting overexpression of the *HPRT1* gene may be resistant to abiraterone and sensitive to several drugs, including tozasertib and teniposide. This study demonstrated that the elevated expression of *HPRT1* gene is correlated with the progression of HNSCC; thus, this gene may serve as a useful indicator for the early detection, risk stratification and targeted therapy of patients with HNSCC.

## Graphical abstract

We report that hypoxanthine phosphoribosyltransferase (HPRT1) expression levels are increased in head and neck squamous cell carcinoma (HNSCC) tissues. *HPRT1* and its associated genes are enriched in DNA replication and cell‐cycle pathways, and *HPRT1* may be a potential indicator for the early detection, risk stratification and targeted therapy of patients with HNSCC.

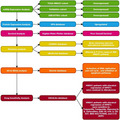

**Abbreviations**
AUC: area under the ROC curve
BP: biological processes
CC: cellular component
CI: confidence interval
COSMIC: catalogue of somatic mutations in cancer
GEO: Gene Expression Omnibus
GO: Gene Ontology
HNSCC: head and neck squamous cell carcinoma
HPA: Human Protein Atlas
HPRT1: hypoxanthine phosphoribosyltransferase
HPV: human papillomavirus
IHC: immunohistochemistry
KEGG: Kyoto Encyclopedia of Genes and Genomes
LSCC: laryngeal squamous cell carcinoma
OS: overall survival
OSCC: oral squamous cell carcinoma
RFS: relapse‐free survival
ROC: receiver operating characteristic
SDHA: succinate dehydrogenase complex flavoprotein subunit A
TCGA: The Cancer Genome Atlas
UCSC: University of California Santa Cruz

Head and neck squamous cell carcinoma (HNSCC) has been introduced as the sixth most common malignancy globally. HNSCC originates from various subsites of the upper aerodigestive tract, such as the oral cavity, larynx, pharynx, paranasal sinuses and nasal cavity [1, 2, 3, 4]. Despite current efforts for the risk stratification and treatment of patients with HNSCC, the overall survival (OS) rate of these patients is unsatisfactory and has not significantly improved over the past decade, which may be because of late‐stage diagnosis. The lack of effective means for clinical applications urgently demands the findings of more applicable biomarkers to improve the earlier detection and targeted therapies of patients with HNSCC [5, 6, 7, 8, 9, 10, 11].

Hypoxanthine phosphoribosyltransferase (*HPRT1*) transcripts the HPRT protein, a transferase enzyme that takes part in the cell cycle through modulation of guanine and inosine production in the salvage pathway [12, 13]. Although the *HPRT1* is broadly used as a housekeeping gene for many expression studies, growing evidence has ascertained the differential expression of *HPRT1* and its imperative role in quickly proliferating cells, such as neoplasms, due to elevated demand for nucleotides synthesis and consequently the *HPRT1* during the cell cycle [14, 15]. For instance, Townsend *et al*. [16] have shown the overexpression of *HPRT1* in colorectal cancer samples compared with healthy tissues. Besides, others have demonstrated that the increased expression of *HPRT1* in endometrial cancer samples was correlated with the survival outcomes of patients [17]. Moreover, Sedano *et al*. [18] also have discovered that up‐regulation of the *HPRT1* gene in tumor tissues could estimate the prognosis of patients with breast cancer. These findings provoked us to appraise the *HPRT1* gene as a probable biomarker for patients with HNSCC.

In this study, we first analyzed the expression patterns of the *HPRT1* gene in HNSCC using bioinformatics and laboratory investigations. We also assessed the diagnostic value and prognostic significance of the *HPRT1* mRNA expression in patients with HNSCC. Besides, we conducted the mutation analysis for the *HPRT1* gene in HNSCC. Finally, we explored those genes closely associated with HPRT1 in HNSCC and then performed functional enrichment analysis for these coexpressed genes to better understand the role of HPRT1 in HNSCC.

## Materials and methods

### Expression analysis using the TCGA–HNSCC cohort

The University of California Santa Cruz (UCSC) Xena browser (https://xenabrowser.net/) is an online web tool for analyzing and visualizing multiomic data and related clinical and phenotypic annotations [19]. We downloaded the mRNA expression levels of the *HPRT1* gene and the information of several clinicopathological parameters of 520 HNSCC samples and 44 normal tissues in The Cancer Genome Atlas (TCGA) cohort from data deposited in the UCSC Xena browser. The clinicopathological features of patients of this cohort are listed in Table 1.

**Table 1:** Clinicopathological features of patients in the TCGA–HNSCC cohort. NA, not available.

Clinicopathological parameter	Category	*n*	Percent (%)
Tissue specimens	HNSCC	520	92.2
	Noncancerous	44	7.8
Age (years)	20–40	24	4.3
	41–60	249	44.1
	61–80	262	46.5
	81–100	27	4.8
	NA	2	0.4
Sex	Female	150	26.6
	Male	414	73.4
Stage	I	27	4.8
	II	74	13.1
	III	81	14.4
	IV	266	47.2
	NA	116	20.6
Grade	G1	62	11.0
	G2	304	53.9
	G3	125	22.2
	G4	7	1.2
	NA	66	11.7

### Expression analysis using the Gene Expression Omnibus database

The Gene Expression Omnibus (GEO) database (https://www.ncbi.nlm.nih.gov/geo/) is a valuable resource of available gene expression data that can be integrated and explored to derive new theories and knowledge [20]. We compared the *HPRT1* mRNA expression in 23 HNSCC and 23 noncancerous tissues using a previously published dataset (GSE107591) [21] obtained from the GEO database. A *P* value <0.05 was considered statistically significant.

### Sample collection

We obtained a total of 45 tumor tissues and paired adjacent normal tissues from 45 patients with HNSCC undergoing routine surgical procedures in the Amir Alam Hospital Complex in Tehran, Iran. It is noteworthy that none of the participants received treatment before the operation. The tissue samples were frozen immediately after surgery in liquid nitrogen and were stored at −80 °C until RNA extraction. The demographic and clinical data of study participants are shown in Table 2. This study was carried out in accordance with the standards of the Declaration of Helsinki and with ethics approval from the Ethics Committee of Hormozgan University of Medical Sciences (IR.HUMS.REC.1399.150). Written informed consent was signed by all patients before involvement in this study.

**Table 2:** Clinicopathological features of patients in the validation cohort.

Clinicopathological parameter	Category	*n*	Percent (%)
Tissue specimens	HNSCC	45	50.0
	Noncancerous	45	50.0
Age (years)	<60	30	66.7
	≥60	15	33.3
Sex	Female	33	73.3
	Male	12	26.7
Stage	I–II	18	40.0
	III–IV	27	60.0
Grade	G1	9	20.0
	G2	15	33.3
	G3	21	46.7

### RNA isolation and quantitative real‐time PCR analysis

Total RNA from the patient’s tissue samples was isolated using TRIzol Reagent (Invitrogen, Carlsbad, CA, USA) based on the manufacturer’s protocol. According to the manufacturer’s protocol, the SuperScript IV Reverse Transcriptase cDNA Synthesis Kit (Roche, Germany) was used for cDNA synthesis. LightCycler 96 Real‐Time PCR was used for the assessment of relative mRNA expression of the *HPRT1* gene using SYBR Premix Ex Taq II (Tli RNaseH Plus; TAKARA, Japan) under the manufacturer’s protocol. Each experiment was conducted in duplicate, and the relative expression level was calculated using the −ddct method normalized with Succinate Dehydrogenase Complex Flavoprotein Subunit A (*SDHA*). The primer sequences used in our study are deposited in Table 3.

**Table 3:** Primer sequences used in this study.

Quantitative real‐time PCR primer	Type of primer	Sequence
HPRT1 (target gene)	Forward primer	5'‐TGGCGTCGTGATTAGTGATG‐3'
HPRT1 (target gene)	Reverse primer	5'‐ACAGAGGGCTACAATGTGATG‑3'
SDHA (reference gene)	Forward primer	5'‐CTTGCCAGGACCTAGAGTTTGT‐3'
SDHA (reference gene)	Reverse primer	5'‐CTCTCCACGACATCCTTCCG‐3’

### Immunohistochemistry staining

The Human Protein Atlas (HPA) database (https://www.proteinatlas.org/pathology) is the largest and most comprehensive repository for proteins in tissues and cells, supplying vital data for assessment of expression profile at a single‐cell resolution [22, 23, 24]. We evaluated the expression of the *HPRT1* gene at protein levels in four HNSCC tissues and one oral mucosa using the immunohistochemistry (IHC) staining data (antibody CAB012200) collected from the HPA database.

### Prognosis analysis

The Kaplan–Meier plotter (http://kmplot.com/analysis) is an open‐access resource that can be exploited to estimate the impact of 54 675 genes on survival outcomes of patients in 21 cancer types [25, 26]. We obtained the information about the prognostic value of the *HPRT1* mRNA expression in HNSCC through this database. A log rank *P* value <0.05 was considered statistically significant.

### Mutation analysis

The Catalogue of Somatic Mutations in Cancer (COSMIC) database (http://cancer.sanger.ac.uk) is a website for studying the influence of somatic mutations in all types of human cancers [27]. We introduced this database to figure out the distribution and substitutions of the *HPRT1* gene mutations in HNSCC. The data have been imported to Excel 2019 software to illustrate the pie chart. Besides, we investigated the genetic alterations of the *HPRT1* gene and their impact on the survival outcomes of patients with HNSCC using the data from the cBio Cancer Genomics Portal (http://cbioportal.org) database, a freely available platform for cancer genomic datasets [28, 29, 30]. A log rank *P* value <0.05 was considered statistically significant.

### Coexpression analysis

We investigated the coexpressed genes of the *HPRT1* gene in HNSCC through the UALCAN database. Genes with extremely low expression (median transcripts per million < 0.5) were removed, and only genes with a |Pearson’s correlation coefficient| ≥ 0.3 were involved. We then evaluated the expression profile of the collected genes in the GEPIA2 database (http://gepia2.cancer‐pku.cn/#index), and only those genes that differentially expressed (*Q* < 0.01, |log2 fold change| > 1) were included for further analyses.

### Functional enrichment analysis

We applied the Enrichr database (http://amp.pharm.mssm.edu/Enrichr) to carry out the Gene Ontology (GO) and Kyoto Encyclopedia of Genes and Genomes (KEGG) pathway analysis for coexpressed genes of the *HPRT1* gene that differentially expressed in HNSCC. The Enrichr database is an extensive platform for curated gene sets and a search engine that gives biological awareness for further biological hypothesis [31]. The GO analysis contained biological processes (BP), molecular function and cellular component (CC). The retrieved terms were imported to Microsoft Excel 2019, and after selecting those terms with an adjusted *P* < 0.01, they were ranked based on the combined score. This index was produced by the Enrichr database and multiplied the log (Fisher’s exact test *P* value) by *z*‐score deviation from the expected rank. The bar chart for the top 10 terms in each category was illustrated using Microsoft Excel 2019.

### Drug sensitivity analysis

To demonstrate whether aberrant expression of the *HPRT1* gene could influence the clinical response to treatment and could be considered a potential biomarker for drug screening, we performed drug sensitivity analysis through the GSCALite database. This database has accumulated 481 and 265 small molecules from the Therapeutics Response Portal and Genomics of Drug Sensitivity in Cancer, respectively, and performs the Spearman’s correlation analysis to evaluate the association of gene expression with drug sensitivity. The positive correlation suggests that the elevated expression of an interesting gene is resistant to the drug and vice versa.

### Statistical analysis

To compare the relative expression of the *HPRT1* gene among different groups, we performed a Student’s *t*‐test or one‐way ANOVA tests as needed. Receiver operating characteristic (ROC) curves were illustrated to demonstrate the diagnostic utility of *HPRT1* mRNA expression for patients with HNSCC. The area under the ROC curve (AUC) closer to 1.0 reflected that the test has the most diagnostic excellence. The maximum Youden index was used as a cutoff point. A *P* value <0.05 was regarded as statistically significant. GraphPad Prism Software, version 9.0.0 (GraphPad Software Inc., La Jolla, CA, USA), was used to perform statistical analysis.

## Results

### The mRNA expression levels of the *HPRT1* gene in HNSCC

**Fig. 1:**
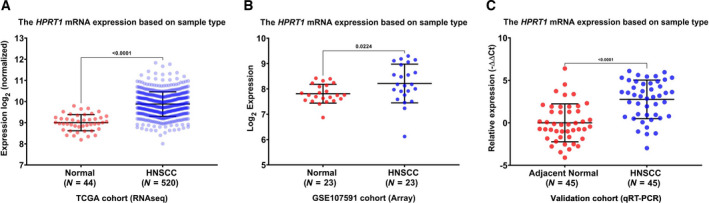
The expression analysis of *HPRT1* mRNA. The comparison of mRNA expression levels of *HPRT1* in 520 cancerous tissues and 44 healthy samples in the TCGA–HNSCC cohort (A). The *HPRT1* mRNA expression levels in 23 HNSCC and 23 normal tissue specimens in the GSE25099 cohort using the GEO database (B). The relative expression of *HPRT1* mRNA in 45 HNSCC samples and adjacent normal tissues in our validation cohort (C). All data are shown as the mean ± standard deviation. *P* < 0.05 was regarded as statistically significant.

We first investigated the transcript expression profiles of the *HPRT1* gene. The RNA sequencing data retrieved from the UCSC Xena browser revealed that the mRNA expression levels of the *HPRT1* gene were higher in HNSCC samples when compared with normal tissues (*P* < 0.0001; Fig. 1A). This was in accordance with our findings obtained from the GSE107591 dataset (*P* = 0.022; Fig. 1B). To confirm the results of our data mining, we assessed mRNA expression levels of the *HPRT1* gene in 90 samples (45 from HNSCC tissues and 45 from adjacent normal tissues) using quantitative real‐time PCR analysis. The results concluded that the transcript levels of the *HPRT1* gene were apparently elevated in HNSCC compared with adjacent normal tissues (*P* < 0.0001; Fig. 1C).

### The relationship between the *HPRT1* mRNA expression and clinicopathological characteristics of patients with HNSCC

**Fig. 2:**
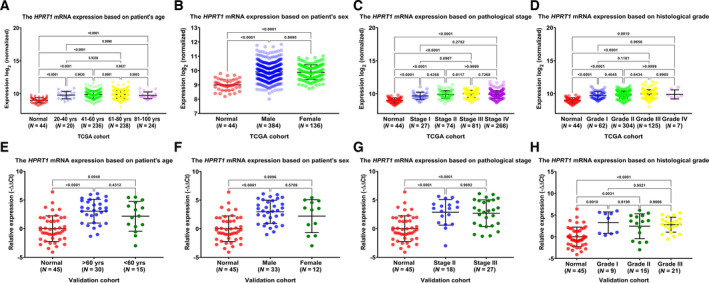
The association between the *HPRT1* mRNA expression levels and clinicopathological features. The correlation of HPRT1 mRNA expression levels with patient’s age (A), sex (B), pathological stage (C) and histological grade (D) in the TCGA–HNSCC cohort. The relationship between the HPRT1 mRNA expression levels and patient’s age (E), sex (F), pathological stage (G) and histological grade (H) in our validation cohort (quantitative real‐time PCR data). Notably, the same normal datasets (*n* = 44 for TCGA–HNSCC cohort and *n* = 45 for validation cohort) have been used multiple times, in A–D and E–H, respectively. All data are shown as the mean ± standard deviation. *P* < 0.05 was regarded as statistically significant.

We further assessed the association of *HPRT1* mRNA expression levels with clinicopathological features of patients in TCGA–HNSCC and our validation cohorts. As presented in Fig. 2A–H, data determined that in comparison with normal control counterparts, the expression levels of the *HPRT1* gene were higher in patients with HNSCC with different age, sex, pathological stage and histological grade. However, further analysis indicated the absence of remarkable variation in mRNA expression levels of the *HPRT1* gene for patients with HNSCC of different ages (Fig. 2A,E), different sexes (Fig. 2B,F), different stages (Fig. 2C,G) and different grades (Fig. 2D,H).

### The diagnostic utility of *HPRT1* mRNA expression for HNSCC

**Fig. 3:**
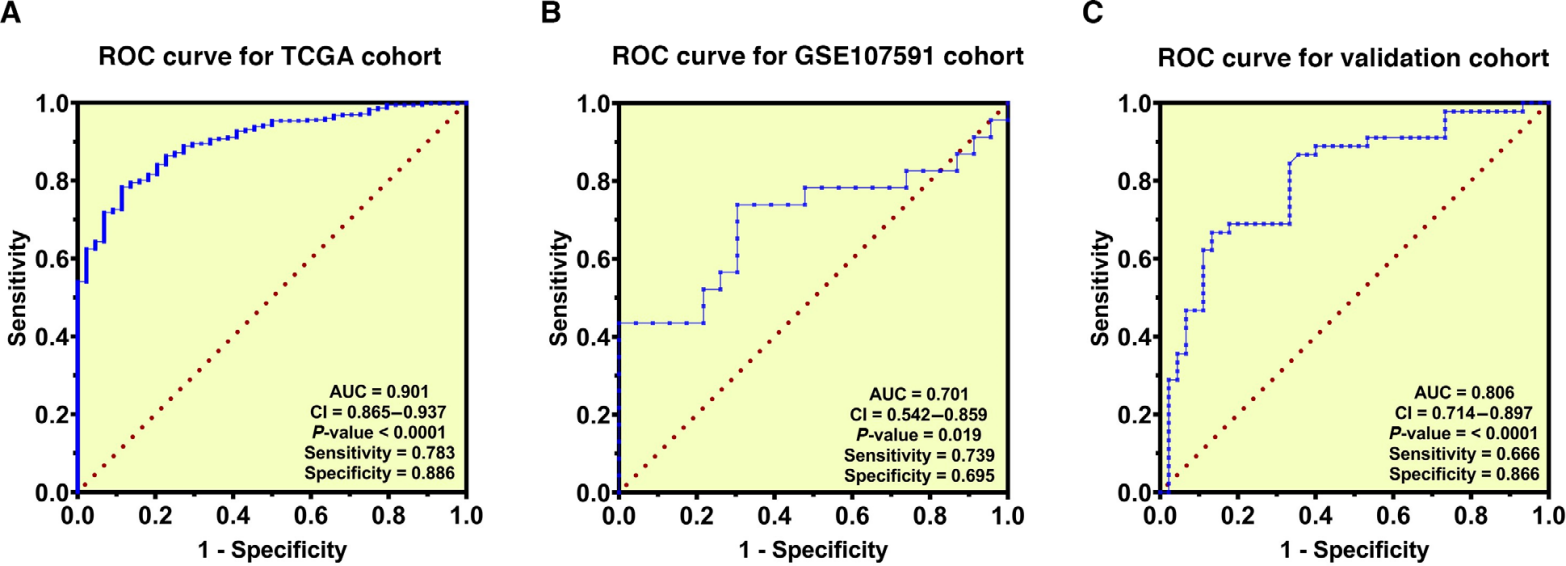
The diagnostic value of *HPRT1* mRNA expression in HNSCC using ROC curves analysis. ROC curves for the TCGA cohort (A). ROC curves for the GSE107591 cohort (B). ROC curves for the validation cohort (quantitative real‐time PCR data) (C).

We appraised the diagnostic merit of *HPRT1* mRNA expression for HNSCC through the calculation of the AUCs. The findings demonstrated the diagnostic capacity of *HPRT1* mRNA expression in patients with HNSCC in TCGA cohort [AUC = 0.901, *P* < 0.0001, 95% confidence interval (CI) = 0.865–0.937; Fig. 3A], GSE107591 cohort (AUC = 0.701, *P* < 0.019, 95% CI = 0.542–0.859; Fig. 3B) and our validation cohort (AUC = 0.806, *P* < 0.0001, 95% CI = 0.714–0.897; Fig. 3C).

### Prognostic value of *HPRT1* mRNA expression for HNSCC

**Fig. 4:**
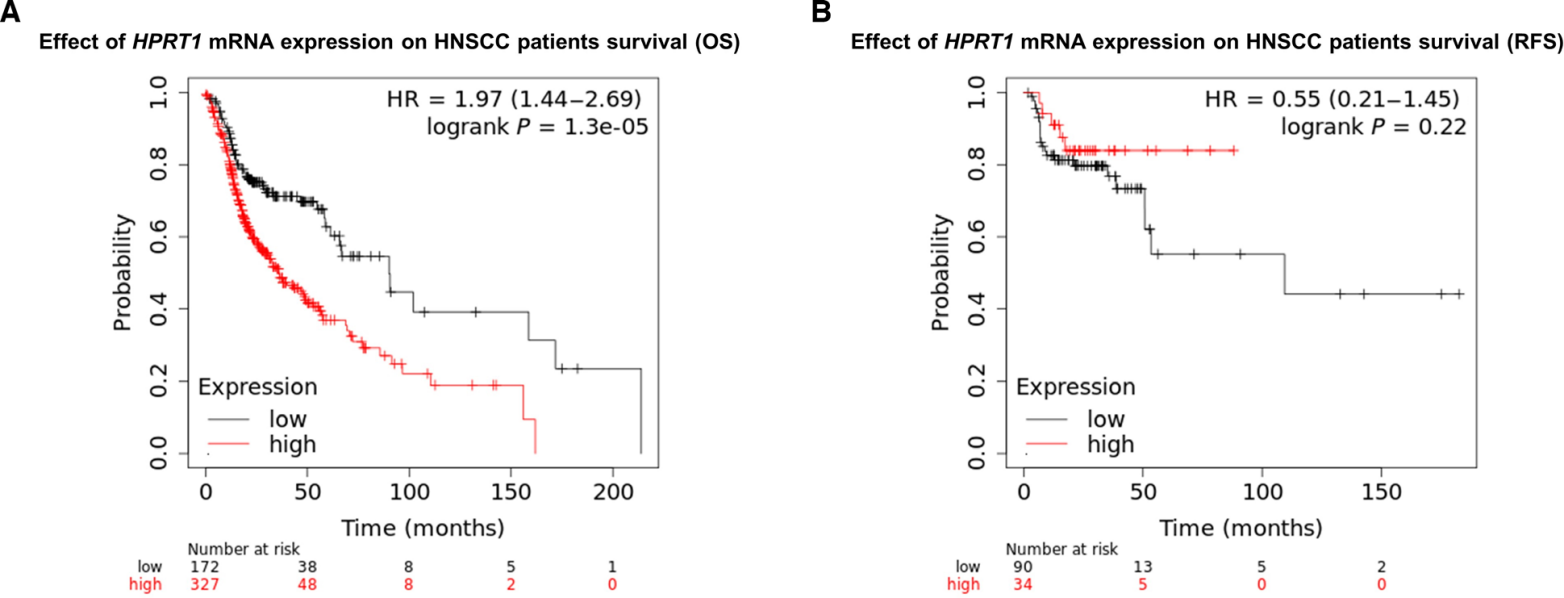
The prognostic significance of *HPRT1* mRNA expression in HNSCC using the data from the Kaplan–Meier Plotter database. The association between the *HPRT1* mRNA expression and OS in patients with HNSCC (A). The relationship between the *HPRT1* mRNA expression and RFS in patients with HNSCC (B). A log rank *P* < 0.05 was considered statistically significant.

We evaluated the prognostic value of *HPRT1* mRNA expression for HNSCC using the Kaplan–Meier Plotter database. The statistics suggested that higher levels of *HPRT1* transcripts were correlated with the poor OS (*P* = 1.3e−5; Fig. 4A). However, there was no significant difference between relapse‐free survival (RFS) in patients with HNSCC with high expression levels of *HPRT1* mRNA compared with those with low expression levels (*P* = 0.22; Fig. 4B).

### The expression levels of the HPRT1 protein in HNSCC

**Fig. 5:**
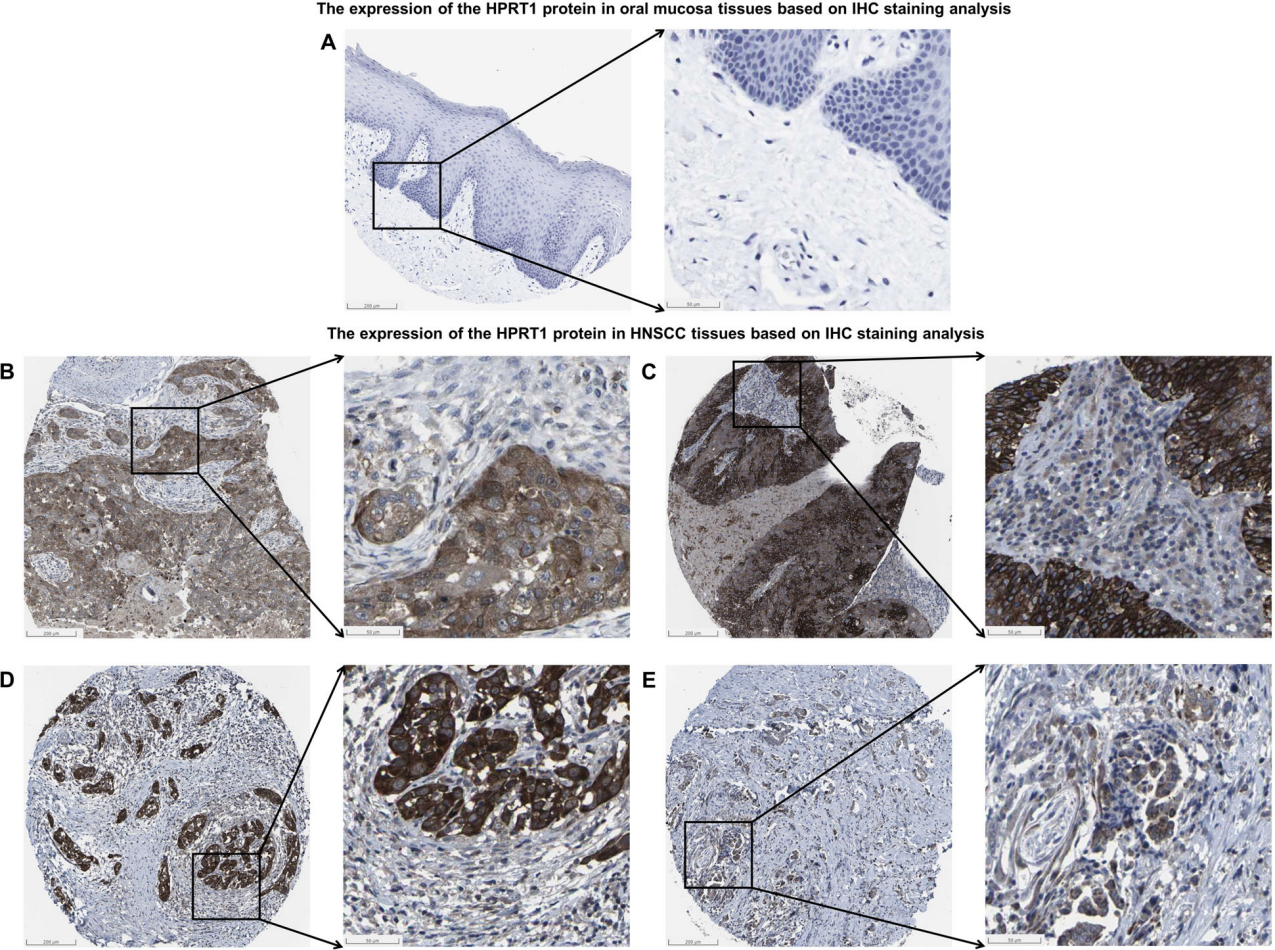
The proteomic analysis of HPRT1 expression in HNSCC using the data from the HPA database. The undetectable expression of HPRT1 protein in normal oral mucosa of a 71‐year‐old male patient, Patient ID: 1711 (A). The medium expression of HPRT1 protein in tumor tissue of a 51‐year‐old male patient with HNSCC, Patient ID: 2608 (B). The high expression of HPRT1 protein in tumor tissue of a 62‐year‐old male patient with HNSCC, Patient ID: 1743 (C). A 50‐year‐old female patient with HNSCC, Patient ID: 1176 (D). A 59‐year‐old female patient with HNSCC, Patient ID: 773 (E). Scale bars, 200 and 50 μm. HR, hazard ratio.

To check the expression levels of the *HPRT1* gene at protein levels, we then acquired the relevant expression data using the IHC staining images from the HPA database. The findings showed negative HPRT1 staining in normal oral mucosa of one patient, and moderate and strong HPRT1 staining in tumor tissues of one and three patients with HNSCC, respectively (Fig. 5A–E). Regarding the distribution, the expression of the HPRT1 protein is mainly noticed in the cytoplasm and cell membrane (Table 4).

**Table 4:** The IHC staining data of HPRT1 protein in one oral mucosa and four HNSCC tissues based on the HPA database.

Patient parameters			Staining with antibody CAB012200			
ID; status	Sex	Age (years)	Staining	Intensity	Quantity	Location
1711; normal	Male	71	Not detectable	Negative	None	None
2608; HNSCC	Male	51	Medium	Moderate	>75%	Cytoplasmic/membranous
1743; HNSCC	Male	62	High	Strong	>75%	Cytoplasmic/membranous
1176; HNSCC	Female	50	High	Strong	>75%	Cytoplasmic/membranous
773; HNSCC	Female	59	High	Strong	25% to 75%	Cytoplasmic/membranous

### Mutation analysis of the *HPRT1* gene

**Fig. 6:**
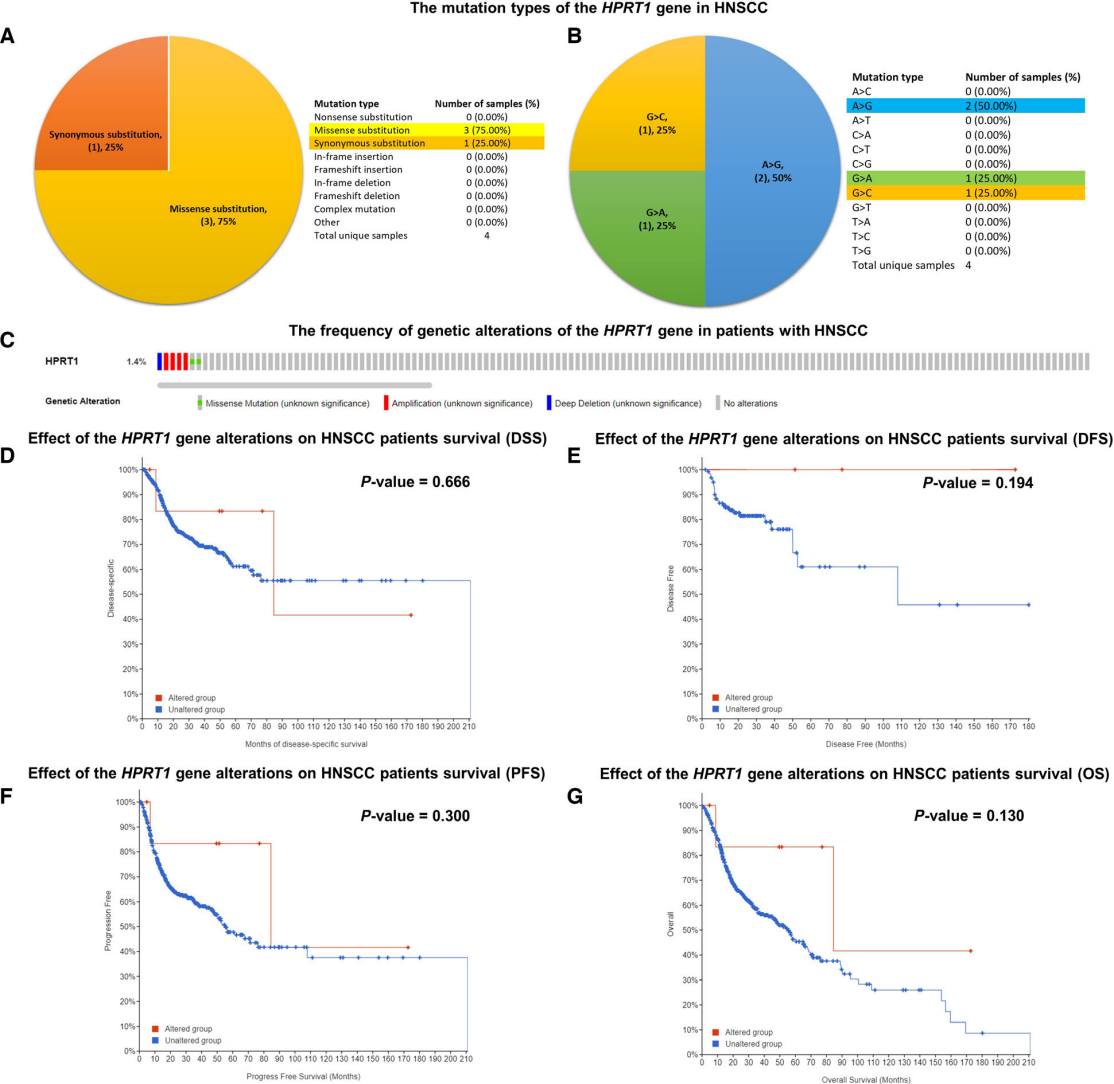
The mutation analysis for the *HPRT1* gene in HNSCC. The distribution and substitutions of mutations in the coding strand of the *HPRT1* gene in HNSCC are based on the COSMIC database (A, B). The genetic alterations of the *HPRT1* gene and their frequencies in patients with HNSCC according to the cBioPortal database (C). The association between the genetic alterations of the *HPRT1* gene and disease‐specific survival (DSS) in patients with HNSCC using the data from the cBioPortal database (D). The relationship between the genetic alterations of the *HPRT1* gene and disease‐free survival (DFS) in patients with HNSCC using the data from the cBioPortal database (E). The correlation between genetic alterations of the *HPRT1* gene and progression‐free survival (PFS) in patients with HNSCC obtained from the cBioPortal database (F). The association between the genetic alterations of the *HPRT1* gene and OS in patients with HNSCC was retrieved from the cBioPortal database (G). A log rank *P* < 0.05 was considered statistically significant.

The mutations of the *HPRT1* gene in HNSCC samples were examined by the COSMIC database. The pie chart depicted that among four mutations in the *HPRT1* gene, three were missense (75.00%) and one was synonymous (25.00%) substitutions (Fig. 6A). There were 50.00% A>G, 25.00% G>A and 25.00% G>C mutations in the *HPRT1* gene coding region (Fig. 6B). We also explored the types of genetic alterations in the *HPRT1* gene and their frequencies in 496 samples from the TCGA–HNSCC cohort using the cBioPortal database. As shown in Fig. 6C, the *HPRT1* gene was altered in 7 (1%) cases of HNSCC, and the amplification was responsible for most changes. Moreover, using the “Survival” tab, we investigated the relationship between genetic alterations of the *HPRT1* gene and survival times in patients with HNSCC. The Kaplan–Meier plot and log rank test uncovered that genetic alterations of the *HPRT1* gene were not associated with the disease‐specific survival (*P* = 0.666; Fig. 6D), disease‐free survival (*P* = 0.194; Fig. 6E), progression‐free survival (*P* = 0.300; Fig. 6F) and OS (*P* = 0.130; Fig. 6G) of patients with HNSCC.

### Demonstration of the dysregulated coexpressed genes of the *HPRT1* gene

**Fig. 7:**
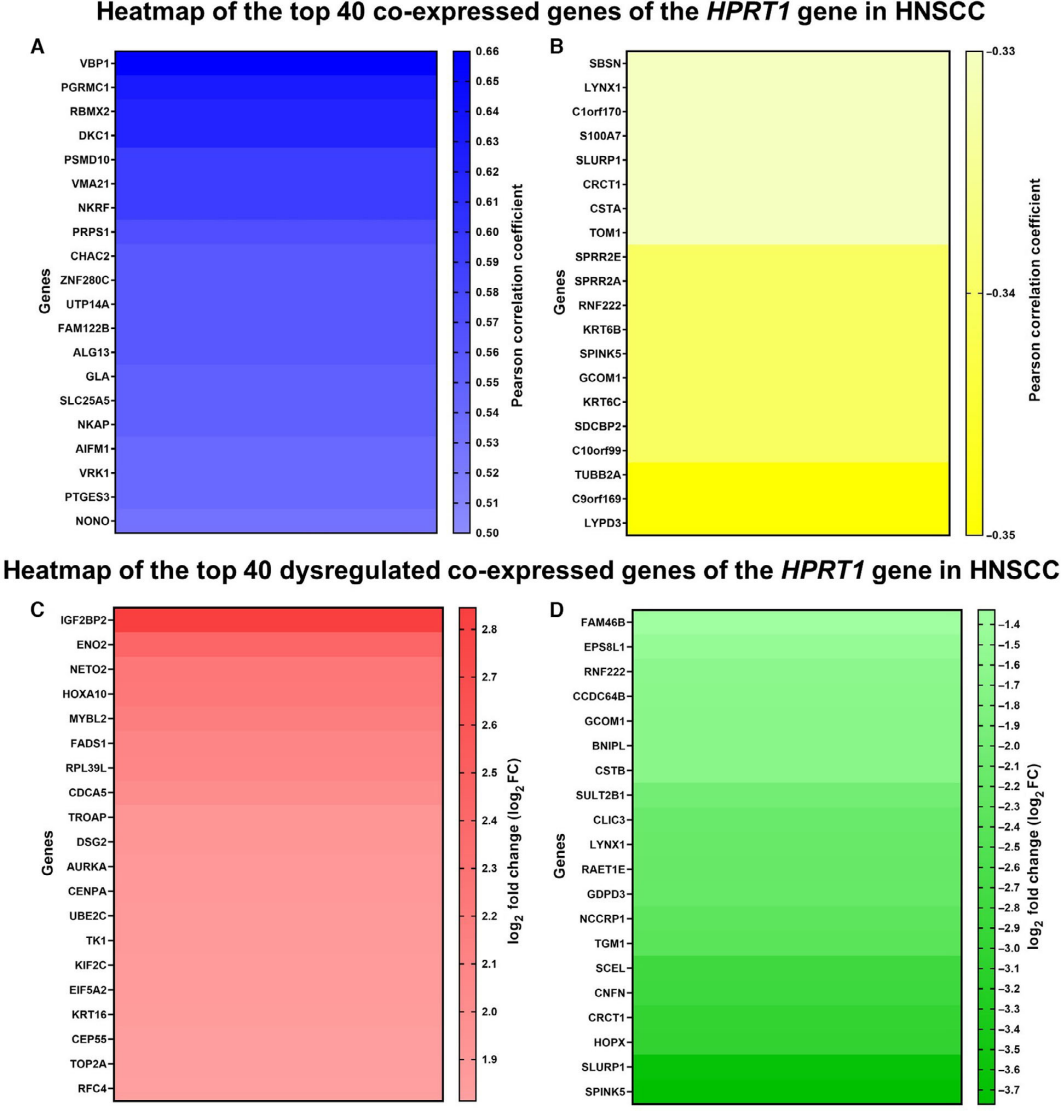
Identification of the dysregulated coexpressed genes of the *HPRT1* gene in HNSCC. Heatmap of the top 20 genes with positive (A) and negative (B) correlation with *HPRT1* in HNSCC. Heatmap of the top 20 up‐regulated (C) and down‐regulated (D) coexpressed genes of the *HPRT1* gene in HNSCC.

To explore the potential roles of the *HPRT1* gene in HNSCC, we first collected the coexpressed genes of the *HPRT1* in HNSCC via the UALCAN database. Our data mining unveiled that 1966 genes were correlated with *HPRT1* in HNSCC. Figure 7A,B shows the top 20 genes with positive and negative correlation with *HPRT1* in HNSCC, respectively. When we assessed the expression value of the acquired genes in the GEPIA2 database, we found that only 268 genes were differentially expressed in HNSCC. Among them, IGF2BP2, ENO2, NETO2, HOXA10 and MYBL2 were the top five up‐regulated (Fig. 7C), and SPINK5, SLURP1, HOPX, CRCT1 and CNFN were the top five down‐regulated coexpressed genes of the *HPRT1* gene in HNSCC (Fig. 7D).

### GO and KEGG pathway analysis

Subsequently, we applied the Enrichr database to conduct the GO and KEGG pathway analysis for dysregulated coexpressed genes of the *HPRT1* gene in HNSCC. The GO analysis unearthed that these genes were enriched in centromere complex assembly (GO:0034508), chromatin remodeling at centromere (GO:0031055), mitotic sister chromatid segregation (GO:0000070), CENP‐A containing nucleosome assembly (GO:0034080), CENP‐A containing chromatin organization (GO:0061641), regulation of attachment of spindle microtubules to kinetochore (GO:0051988), histone exchange (GO:0043486), sister chromatid segregation (GO:0000819), regulation of metaphase/anaphase transition of cell cycle (GO:1902099) and mitotic spindle elongation (GO:0000022) in BP (Table 5); and in histone kinase activity (GO:0035173), histone serine kinase activity (GO:0035174), 3'–5’ DNA helicase activity (GO:0043138), DNA clamp loader activity (GO:0003689), protein–DNA loading ATPase activity (GO:0033170), DNA helicase activity (GO:0003678), single‐stranded DNA‐dependent ATPase activity (GO:0043142), peptide disulfide oxidoreductase activity (GO:0015037), DNA‐dependent ATPase activity (GO:0008094) and four‐way junction DNA binding (GO:0000400) in molecular function (Table 6); and in condensed nuclear chromosome kinetochore (GO:0000778), condensed chromosome, centromeric region (GO:0000779), condensed nuclear chromosome, centromeric region (GO:0000780), spindle midzone (GO:0051233), condensed chromosome kinetochore (GO:0000777), chromosome, centromeric region (GO:0000775), spindle microtubule (GO:0005876), spindle (GO:0005819), Alpha DNA polymerase:primase complex (GO:0005658) and chromosomal region (GO:0098687) in CC (Table 7). The KEGG pathway analysis also indicated that these genes were enriched in DNA replication, cell cycle, homologous recombination, Fanconi anemia pathway, p53 signaling pathway, progesterone‐mediated oocyte maturation, mismatch repair, oocyte meiosis, one carbon pool by folate and cellular senescence pathways (Table 8).

**Table 5:** The top 10 GO biological process terms for the *HPRT1*‐correlated genes with differential expression in HNSCC.

Term	*P* value	Adjusted *P* value	Combined score
Centromere complex assembly (GO:0034508)	1.32E−17	3.74E−15	1911.778
Chromatin remodeling at centromere (GO:0031055)	4.87E−15	7.70E−13	1516.523
Mitotic sister chromatid segregation (GO:0000070)	7.06E−23	3.35E−20	1489.400
CENP‐A containing nucleosome assembly (GO:0034080)	9.25E−14	9.40E−12	1327.506
CENP‐A containing chromatin organization (GO:0061641)	9.25E−14	9.40E−12	1327.506
Regulation of attachment of spindle microtubules to kinetochore (GO:0051988)	1.01E−7	2.94E−6	1203.491
Histone exchange (GO:0043486)	2.58E−14	3.66E−12	1199.678
Sister chromatid segregation (GO:0000819)	2.13E−13	1.90E−11	1167.513
Regulation of metaphase/anaphase transition of cell cycle (GO:1902099)	2.15E−6	4.30E−5	971.566
Mitotic spindle elongation (GO:0000022)	1.83E−7	4.92E−6	965.806

**Table 6:** The top 10 GO molecular function terms for the *HPRT1*‐correlated genes with differential expression in HNSCC.

Term	*P* value	Adjusted *P* value	Combined score
Histone kinase activity (GO:0035173)	5.11E−8	3.05E−6	1568.152
Histone serine kinase activity (GO:0035174)	1.09E−6	3.59E−5	1363.233
3'–5' DNA helicase activity (GO:0043138)	5.00E−7	2.13E−5	677.475
DNA clamp loader activity (GO:0003689)	8.09E−5	1.10E−3	524.070
Protein–DNA loading ATPase activity (GO:0033170)	8.09E−5	1.10E−3	524.070
DNA helicase activity (GO:0003678)	2.39E−9	2.32E−7	436.665
Single‐stranded DNA‐dependent ATPase activity (GO:0043142)	1.46E−5	3.10E−4	414.475
Peptide disulfide oxidoreductase activity (GO:0015037)	1.90E−4	2.36E−3	317.626
DNA‐dependent ATPase activity (GO:0008094)	3.11E−9	2.32E−7	269.318
Four‐way junction DNA binding (GO:0000400)	5.13E−5	8.49E−4	245.0186

**Table 7:** The top 10 GO CC terms for the *HPRT1*‐correlated genes with differential expression in HNSCC.

Term	*P* value	Adjusted *P* value	Combined score
Condensed nuclear chromosome kinetochore (GO:0000778)	2.32E−11	3.52E−10	3226.434
Condensed chromosome, centromeric region (GO:0000779)	2.24E−16	9.35E−15	2369.685
Condensed nuclear chromosome, centromeric region (GO:0000780)	4.83E−9	5.38E−8	1436.064
Spindle midzone (GO:0051233)	9.25E−14	2.20E−12	1327.506
Condensed chromosome kinetochore (GO:0000777)	6.42E−11	8.93E−10	1289.637
Chromosome, centromeric region (GO:0000775)	6.03E−17	3.36E−15	1277.699
Spindle microtubule (GO:0005876)	2.00E−15	6.69E−14	1045.629
Spindle (GO:0005819)	3.11E−26	5.20E−24	1008.086
Alpha DNA polymerase:primase complex (GO:0005658)	4.67E−5	2.79E−4	739.5584
Chromosomal region (GO:0098687)	3.86E−15	1.07E−13	688.1082

**Table 8:** The top 10 KEGG terms for the *HPRT1*‐correlated genes with differential expression in HNSCC.

Term	*P* value	Adjusted *P* value	Combined score
DNA replication	9.47E−13	7.15E−11	930.412
Cell cycle	3.18E−16	4.81E−14	540.898
Homologous recombination	6.28E−8	3.16E−6	303.411
Fanconi anemia pathway	5.88E−7	1.27E−5	188.182
p53 signaling pathway	5.12E−7	1.27E−5	156.536
Progesterone‐mediated oocyte maturation	9.41E−8	3.55E−6	153.973
Mismatch repair	2.32E−4	3.50E−3	131.078
Oocyte meiosis	1.26E−7	3.79E−6	128.813
One carbon pool by folate	2.32E−3	3.18E−2	79.364
Cellular senescence	6.36E−5	1.20E−3	48.705

### Drug sensitivity analysis

**Fig. 8:**
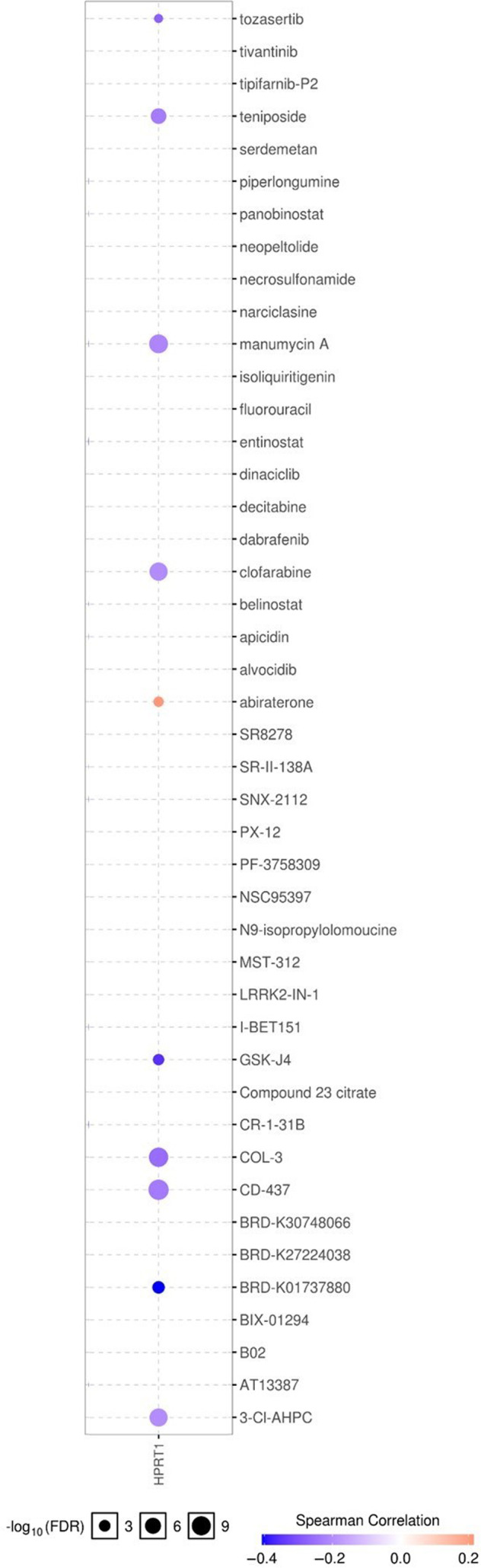
The drug sensitivity analysis for the *HPRT1* gene. The data from the GSCALite database represent the correlation of the *HPRT1* gene expression and drug sensitivity. The negative correlation determines that patients with cancer with overexpression of the *HPRT1* gene are sensitive to the drug and vice versa.

We investigated the correlation between the expression profile of the *HPRT1* gene with drug sensitivity through the GSCALite database. The results have suggested that patients with cancer with high expression of the *HPRT1* gene probably were sensitive to tozasertib, teniposide, manumycin A, clofarabine, GSK‐J4, COL‐3, CD‐437, BRD‐K01737880 and 3‐Cl‐AHPC and resistant to abiraterone (Fig. 8).

## Discussion

The *HPRT1* aberrant expression has been documented in several cancers [15]. Nevertheless, the relationship between the *HPRT1* gene and HNSCC is unknown. To our knowledge, our report is the first to investigate the expression of the *HPRT1* gene at transcriptomic and proteomic levels, as well as its genetic alterations, diagnostic and prognostic merit, and probable functions in the head and neck squamous cancer. We hope that the findings of the present research advance the current knowledge, enhance therapeutic targets and strengthen the accuracy of diagnosis and prognosis for patients with HNSCC.

This study initially employed the data from the TCGA–HNSCC cohort and GSE107591 dataset to demonstrate the *HPRT1* mRNA expression in HNSCC. The results showed that the *HPRT1* mRNA expression was significantly up‐regulated in cancer tissues compared with healthy control samples, which was consistent with the findings of our quantitative real‐time PCR analysis for 45 paired HNSCC and normal tissues. We also assessed the expression levels of the *HPRT1* gene in patients with HNSCC with different sex, age, pathological stage and histological grades in TCGA–HNSCC and our validation cohorts. The sex‐based and age‐based differences have contributed to the clinical presentations and prognosis of patients with HNSCC, respectively [32, 33, 34, 35]. The sex‐based analysis for both cohorts indicated the significant overexpression of *HPRT1* gene level in tumor samples of both sexes compared with the normal tissues. However, there were no statistically significant differences between transcript levels of the *HPRT1* gene in cancerous tissues of males and females. Besides, we observed no significant changes in the *HPRT1* mRNA expression levels in the tumor tissues of patients with HNSCC of different ages in both cohorts. These may reflect that the patients’ sex and age probably did not influence the expression levels of *HPTR1* mRNA in cancer tissues. We also discovered that the *HPRT1* mRNA expression levels were remarkably elevated in advanced pathological stages and histological grades of HNSCC tissues in comparison with healthy control subjects in both cohorts. These observations suggested that the elevated expression levels of the *HPRT1* gene may take part in the pathogenesis of HNSCC. The IHC staining images from the HPA database have revealed the medium and high expression levels of the HPRT1 protein in HNSCC tissues. Altogether, these findings unearthed that expression of the *HPRT1* gene was significantly increased in HNSCC tissues.

One of the most difficult challenges about patients with HNSCC is the time and procedure of screening and diagnosis. It has been declared that the delayed diagnosis and treatment of patients with HNSCC aggravate the prognosis outcomes and increase the undesired morbidity and mortality of patients with cancer. Hence findings of new biomarkers would boost the clinical outcomes of patients with HNSCC [36, 37, 38, 39]. In the present research, we carried out the ROC curves analysis to appraise the diagnostic utility of the *HPRT1* mRNA expression levels for HNSCC. The data from TCGA (AUC = 0.901), GSE107591 (AUC = 0.701) and our validation cohort (AUC = 0.806) concluded that expression levels of the *HPRT1* gene could be considered as a helpful marker for the diagnosis of patients with HNSCC. Moreover, the prognosis data obtained from the Kaplan–Meier Plotter disclosed that the higher expression levels of the *HPRT1* gene in cancer tissues were significantly correlated with inferior survival time of patients with HNSCC, in line with the findings of previous studies that suggested the dysregulation of the *HPRT1* expression was associated with poor survival outcomes of breast and endometrial cancers [17, 18]. In summary, these results may indicate the great value of the *HPRT1* tissue expression for diagnosis and risk stratification of patients with HNSCC.

It is widely accepted that the accumulation of genetic alterations plays a causal role in the tumorigenesis of HNSCC [40]. The further assessments using the COSMIC and cBioPortal databases unveiled that genetic alterations of the *HPRT1* gene were rare in patients with HNSCC (7/496), and the missense mutations were responsible for the most types of genetic aberration in the *HPRT1* gene coding strand. Besides, the genetic alterations of the *HPRT1* gene were not associated with survival outcomes of patients with HNSCC.

Finding dysregulated coexpressed genes of the *HPRT1* gene in HNSCC may lead to understanding how HPRT1 and its correlated genes help the progression of HNSCC. In the present research, we uncovered that *HPRT1* and its coexpressed genes that are differentially expressed in HNSCC probably were enriched in the DNA replication, cell cycle, homologous recombination, Fanconi anemia, p53 signaling and mismatch repair pathways. When we checked the included coexpressed genes and their expression value in DNA replication (RFC5, PRIM2, FEN1, POLA2, RNASEH2A, RFC4, MCM7, RPA3, POLE2, MCM3, MCM6), cell cycle (MCM7, PLK1, BUB1B, TTK, CDC6, CDC25C, CDC20, CCNA2, CCNB2, CCNB1, CDC45, CDK4, CHEK2, CHEK1, CDK2, CDK1, MCM3, MCM6, BUB1, MAD2L1), homologous recombination (BLM, RAD51, EME1, RPA3, XRCC3, RAD54L, BRCA1, RAD54B), Fanconi anemia (FANCI, BLM, RAD51, EME1, UBE2T, RPA3, FANCA, BRCA1), p53 signaling (CCNB2, CCNB1, RRM2, CDK4, CHEK2, CHEK1, CDK2, CDK1, GTSE1) and mismatch repair (RFC5, RFC4, EXO1, RPA3) pathways, we found that all of them were significantly up‐regulated in HNSCC. Previous studies have documented the homologous recombination and Fanconi anemia pathways deficiency in HNSCC [41, 42]. Besides, further investigations showed several genes in DNA replication, and cell‐cycle pathways were duplicated in the p53 signaling (CCNB2, CCNB1, CDK4, CHEK2, CHEK1, CDK2, CDK1) and mismatch repair (RFC5, RFC4, RPA3) pathways. Moreover, several pieces of evidence have reported that up‐regulation of DNA replication and cell‐cycle pathways genes in HNSCC [43, 44, 45]. Others have proposed that overexpression of the RRM2, GTSE1 and EXO1 genes in HNSCC have active roles in tumor progression and inhibition of the apoptosis pathway [46, 47, 48, 49]. Some researchers have suggested that the *HPRT1* gene, as a crucial component of the purine salvage pathway, plays a role in mediating the proliferation, autophagy and apoptosis‐related processes in cancer cells [50, 51, 52]. *IGF2BP2* and *SPINK5* were the top up‐regulated and down‐regulated coexpressed genes of the *HPRT1* gene in HNSCC. Deng *et al*. [53] have determined that *IGF2BP2* is a prognostic gene and has vital roles in the progression of HNSCC. Others also have recognized *SPINK5* as a novel tumor suppressor that suppresses migration and invasion of HNSCC cells [54, 55]. These findings imply that *HPRT1* and its associated gene likely mediate the progression of HNSCC through activation of the DNA replication and cell cycle and inhibition of apoptosis. Finally, the data obtained from the GSCALite database indicated that patients with increased expression of the *HPRT1* gene may not respond to treatment with abiraterone very well and hopefully are responsive to therapies that consist of tozasertib, teniposide, manumycin A, clofarabine, GSK‐J4, COL‐3, CD‐437, BRD‐K01737880 and 3‐Cl‐AHPC.

Our study had some limitations:

1. As we stated in the introduction, HNSCCs are heterogeneous and can develop at different locations, such as the oral cavity, pharynx and larynx [4]. When we aimed to classify patients with HNSCC of TCGA, GSE107591 and our validation cohorts into oral or laryngeal squamous cell carcinoma, the number of patients in each group was few, and we could not perform meaningful statistical analyses. Therefore, we decided to consider all of them as HNSCC.
2. Although the expression analysis for the TCGA cohort demonstrated the absence of significant differences in the expression levels of the *HPRT1* gene for patients with HNSCC who were human papillomavirus (HPV) positive in comparison with those with HPV negative (data not shown), we could not find any data about the HPV history of patients with HNSCC in GSE107591 and our validation cohorts.
3. The samples in the HPA database were few.
4. Although we determined that the expression levels of the *HPRT1* gene were increased in HNSCC, the underlying mechanisms for this phenomenon remained unclear.
5. In this report, we uncovered that the *HPRT1* gene and its dysregulated coexpressed genes likely mediate HNSCC progression through the cell cycle and apoptosis‐related pathways. However, the exact function of the *HPRT1* gene in these pathways remained ambiguous.
6. Except for mRNA expression analysis, other findings were not confirmed with reliable laboratory analysis, and we just focused on bioinformatics investigations. Relying on bioinformatics assessments may induce deviations.

Therefore, additional studies with appropriate sample size are required to explore: (a) the expression pattern of the *HPRT1* gene in HNSCCs, such as oral squamous cell carcinoma (OSCC) ‐and laryngeal squamous cell carcinoma (LSCC); (b) the association between the expression levels of the *HPRT1* gene and HPV status of patients with HNSCC; (c) the potential molecular etiologies for up‐regulation of the *HPRT1* gene in HNSCC; and (d) the exact underlying mechanisms in which the *HPRT1* gene promotes the progression of HNSCC.

## Conclusions

Overall, the findings of this research indicated that overexpression of *HPRT1* is significantly correlated with the progression of HNSCC. Besides, the observed up‐regulation of the *HPRT1* expression in tumor tissues may be a valuable biomarker for diagnosis, prognosis and targeted treatment of patients with HNSCC. However, further investigations and clinical trials are mandatory to elucidate the involvement of *HPRT1* in HNSCC.

## Conflict of interest

The authors declare no conflict of interest.

## Author contributions

NS collected the patient’s samples and laboratory materials. FH, MA and MEK performed the laboratory experiments. MA and NS conducted the data extraction and statistical analysis and initially drafted the paper. PM and NS contributed to the design of the study and supervised all investigations. NS, PM, SP, SR and LH edited the manuscript. MA, SP, NS, SMA and PM checked the final version of the manuscript. All authors have contributed to, read and approved the final manuscript for submission.

## Data Availability

The data of TCGA were obtained from the website of the UCSC Xena browser (https://xenabrowser.net/). The data of the GSE107591 dataset were downloaded from the website of the GEO database (https://www.ncbi.nlm.nih.gov/geo/). The data of the validation cohort used for this research are available from the corresponding author upon request.
